# Iodate reduction by marine aerobic bacteria

**DOI:** 10.3389/fmicb.2024.1446596

**Published:** 2024-09-18

**Authors:** Ken Kine, Shigeki Yamamura, Seigo Amachi

**Affiliations:** ^1^Graduate School of Horticulture, Chiba University, Matsudo, Japan; ^2^Regional Environment Conservation Division, National Institute for Environmental Studies, Tsukuba, Japan

**Keywords:** iodate reduction, iodide production, marine aerobic bacteria, *Roseovarius azorensis*, *idrABP_1_P_2_*

## Abstract

Iodate reductase (Idr) gene cluster (*idrABP_1_P_2_*) is involved in bacterial iodate (IO_3_^−^) respiration under anaerobic conditions. Putative *idr* gene clusters are present in both anaerobic and aerobic bacteria; however, the specific physiological roles of *idr* genes in aerobic bacteria remain unclear. Therefore, in this study, three marine aerobic bacteria with putative *idr* gene clusters (*Roseovarius azorensis*, *Notoacmeibacter marinus*, and *Aliiroseovarius sediminilitoris*) were grown in the presence of iodate to determine whether they can reduce iodate to iodide (I^−^). All tested bacteria almost completely reduced 2 mM iodate under static conditions but only reduced 0.1–0.5 mM iodate under shaking conditions. Moreover, the washed cell suspension of *R. azorensis* reduced iodate only when the cells were pre-grown statically in the presence of iodate. Transcriptional analysis revealed that the expression levels of *idrA*, *idrB*, *idrP_1_*, and *idrP_2_* genes were upregulated in *R. azorensis* when the cells were grown statically in the presence of iodate. Specifically, *idrA* expression was induced by 0.1 μM iodate and was up to 14-fold higher compared to that of the non-iodate control. These results suggest that marine aerobic bacteria reduce iodate under oxygen-limited conditions, and that this capacity is induced by environmentally relevant levels of iodate in seawater. Our results suggest that marine aerobic bacteria contribute to iodide production in marine surface waters, thereby affecting the global iodine cycling and ozone budget.

## 1 Introduction

In marine environments, iodine mainly exists in inorganic forms, such as iodate (IO_3_^−^) and iodide (I^−^) ions (Wong, 1991; Luther, 2023). These inorganic iodine species are ubiquitously distributed in seawater at concentrations of 0.4–0.5 μM. Iodate is thermodynamically more stable in oxidized seawater (Sillen, 1961). However, iodide-rich water sources, such as the oxygen minimum zone (OMZ) of Arabian Sea (Farrenkopf et al., 1997; Farrenkopf and Luther, 2002) and hypersaline anoxic brine in the Orca Basin, Gulf of Mexico (Wong et al., 1985), are also observed. High concentrations of iodide are also observed in surface water (Tian and Nicolas, 1995; Campos et al., 1996; Tian et al., 1996; Moriyasu et al., 2023). Moriyasu et al. (2023) recently reported a hot zone of iodide comprising up to 50% of the total iodine concentration in tropical surface waters.

Iodide in marine surface water plays an important role in atmospheric iodine photochemistry and has a significant impact on the global ozone (O_3_) budget (Carpenter et al., 2021). Iodide in seawater reacts with gaseous ozone to form volatile I_2_ and hypoiodous acid (Jones et al., 2010; Carpenter et al., 2013). These volatile iodine compounds undergo photolysis in the atmosphere, releasing iodine atoms (I), which react with ozone to form the IO radical that plays a key role in the formation of cloud condensation nuclei, thus impacting the regional climate (O’Dowd et al., 2002; Saiz-Lopez et al., 2012). The reaction between iodide and ozone at the sea surface is the primary source of atmospheric iodine and the primary sink for atmospheric ozone (MacDonald et al., 2014). Atmospheric iodine reaches the land via wet (rainfall) and dry deposition, where it is taken up by animals and plants (Fuge and Johnson, 2015). Dietary iodine deficiency causes endemic goiter and congenital hypothyroidism (Chaker et al., 2022; Dijck-Brouwer et al., 2022). Therefore, iodide levels in marine surface water significantly influence the public health.

As the iodide concentration in seawater often correlates with chlorophyll fluorescence (Moriyasu et al., 2023; Jones et al., 2024), many studies investigated iodate reduction by microalgae (phytoplankton). Many microalgal cultures are capable of iodate reduction, including growth-associated and senescence-associated reduction (Wong et al., 2002; Chance et al., 2007; Bluhm et al., 2010; van Bergeijk et al., 2016; Hepach et al., 2020). Nitrate reductase is suggested as the key enzyme catalyzing iodate reduction (Tsunogai and Sase, 1969); however, some studies suggest the involvement of other enzymes in this process (Waite and Truesdale, 2003; Mok et al., 2018; Hepach et al., 2020). Recently, bacterial iodate reduction has attracted considerable attention (Yeager et al., 2017). Some bacteria act as iodate-respiring prokaryotes and dissimilatorily reduce iodate as a terminal electron acceptor (Yamazaki et al., 2020; Reyes-Umana et al., 2022a). Iodate-respiring bacteria, such as *Pseudomonas* sp. SCT and *Denitromonas* sp. IR-12, possess gene cluster *idrABP_1_P_2_* in their genome that forms an operon-like structure. Transcription of the *idrABP_1_P_2_* cluster is specifically upregulated under iodate-respiring conditions (Yamazaki et al., 2020). Notably, *idrA* knockout mutant of *Denitromonas* sp. IR-12 does not grow in the presence of iodate (Reyes-Umana et al., 2022a). The *idrA* gene encodes the catalytic subunit of a novel dimethyl sulfoxide reductase family protein containing a molybdenum cofactor and [3Fe–4S] cluster. The *idrB* gene encodes the electron-transfer subunit containing the [2Fe–2S] cluster. Both *idrP_1_* and *idrP_2_* encode di-heme proteins closely related to cytochrome *c* peroxidase, but their functions remain unclear.

The *idrABP_1_P_2_* cluster is widely distributed in the genomes of anaerobic, facultative anaerobic, and aerobic bacteria (Yamazaki et al., 2020; Reyes-Umana et al., 2022a). The *idrABP_1_P_2_* cluster is present in various aerobic Alphaproteobacteria isolated from surface seawater (Supplementary Figure S1). Alphaproteobacteria are predominantly found together with Gammaproteobacteria and Cyanobacteria in the surface waters of Mediterranean Sea, Black Sea, South Atlantic Ocean, and Northwestern Pacific Ocean (Haro-Moreno et al., 2018; Cabello-Yeves et al., 2021; Coutinho et al., 2021; Shih et al., 2023). In this study, three aerobic marine Alphaproteobacteria with the *idrABP_1_P_2_* cluster in their genomes, namely *Roseovarius azorensis* (Rajasabapathy et al., 2014), *Notoacmeibacter marinus* (Huang et al., 2017), and *Aliiroseovarius sediminilitoris* (Park et al., 2015), were grown with iodate under various conditions to determine their iodate reduction capacity. *R. azorensis* was selected as the representative bacterium, and the effects of environmental factors on its iodate-reducing ability and transcription of *idrABP_1_P_2_* genes were investigated. Furthermore, iodate concentration necessary to induce *idrA* transcription was examined and whether this concentration was comparable to the iodine levels in seawater was assessed. The possible physiological roles of iodate reduction by aerobic marine bacteria were also discussed.

## 2 Materials and methods

### 2.1 Growth conditions and culture media

*R. azorensis* KCTC 32421^T^ (Rajasabapathy et al., 2014), *N. marinus* KCTC 52427^T^ (Huang et al., 2017), and *A. sediminilitoris* KCTC 23959^T^ (Park et al., 2015) were purchased from Korean Collection for Type Cultures (Jeonbuk, Korea). They were routinely subcultured aerobically at 30°C on the marine agar 2216 medium (Becton Dickinson, Sparks, MD). For iodate reduction, the cells were cultured in a 100 mL Erlenmeyer flask containing 25 mL of marine broth 2216 (Becton Dickinson) supplemented with 2 mM potassium iodate (Fujifilm Wako Chemicals, Osaka, Japan). The flask was sealed with a silicone septum and incubated at 30°C under static or shaking conditions at 180 rpm.

### 2.2 Iodate reduction by washed cell suspension

*R. azorensis* was grown under static or shaking conditions in the presence or absence of 2 mM iodate as described above. After 3 days, the cells were collected and washed twice with the washing buffer containing 20 mM Tris-HCl (pH 8.0), 330 mM NaCl, 30 mM MgCl_2_·6H_2_O, 13 mM MgSO_4_·7H_2_O, 12 mM CaCl_2_·2H_2_O, and 7 mM KCl. The washed cells were resuspended in the same buffer to yield an optical density at 600 nm (OD_600_) of 1.0. The washed cell suspension (20 mL) was dispensed into the 100 mL Erlenmeyer flask or 60 mL serum bottle under N_2_ gas stream. Glucose (5 mM) and iodate (1 mM) were added to the cell suspension as the electron donor and acceptor, respectively. The flask was sealed with a silicone septum and incubated aerobically with shaking as described above. The serum bottle was sealed with a thick butyl rubber stopper and aluminum cap and incubated anaerobically under static conditions.

### 2.3 Preparation of crude cell extract and enzyme assay

To prepare the crude extract, the cells were grown statically with 2 mM iodate for 3 days, collected, washed, and resuspended in the washing buffer. The cells were disrupted via sonication (Q500 Sonicator; Qsonica, Newtown, CT, United States), followed by centrifugation (17,000 × g, 30 min, 4°C) to remove the cell debris, and the supernatant was used as the crude enzyme. Then, iodate reductase (Idr) activity was assayed using a spectrophotometer with reduced methyl viologen (MV) as an electron donor, as previously described (Yamazaki et al., 2020). The detection limit of the enzyme assay was approximately 0.1 U mg^−1^.

### 2.4 Electrophoresis, activity staining, and liquid chromatography-tandem mass spectrometry analyses

Electrophoresis was performed in two steps to separate the proteins present in the crude extract (Yamazaki et al., 2020). First, non-denaturing gel electrophoresis was performed at 4°C using 10% polyacrylamide gel in 25 mM Tris-glycine buffer (pH 8.3), excluding both the reducing agent and SDS. Following non-denaturing electrophoresis, the gel was incubated in a nitrogen atmosphere with 20 mM Tris-HCl (pH 6.8) containing 0.3 mM MV, 12 mM iodate, and 10 mM dithionite. Then, the clear band (active band) was excised from the gel, boiled with 2% SDS and 5% 2-mercaptoethanol for 5 min for complete denaturation, and subjected to sodium dodecyl sulfate-polyacrylamide gel electrophoresis (SDS-PAGE). A Protein Molecular Weight Marker (Takara, Otsu, Japan) was used as the standard. Proteins were visualized by staining with Coomassie brilliant blue R-250, and the selected bands were excised, trypsin-digested, and subjected to liquid chromatography-tandem mass spectrometry (LC-MS/MS) analysis, as previously described (Shevchenko et al., 1996).

### 2.5 Transcriptional analysis of *idr* genes

All manipulations were performed as previously described (Yamazaki et al., 2020). RNA was obtained from the static and shaking cultures in the presence (0.1–1,000 μM) or absence of iodate. Cells in the late exponential growth phase were collected, and pellets were stored at −80°C. Total RNA was extracted using the RNeasy Miniprep kit (QIAGEN, Hilden, Germany) and treated with the TURBO DNA-free kit (Ambion, Carlsbad, CA, United States) to remove the residual DNA. cDNA was synthesized using the SuperScript First-Strand Synthesis System for reverse transcription-polymerase chain reaction (RT-PCR; Invitrogen, Carlsbad, CA, United States). Quantification of *idrA*, *idrB*, *idrP_1_*, and *idrP_2_* in cDNA samples was performed using quantitative PCR (qPCR). The *16S rRNA* gene was quantified in the cDNA samples to normalize the qRT-PCR data. As shown in Supplementary Table S3, new primers were designed for qPCR analysis. SYBR Green detection was performed for qPCR assays using the StepOnePlus instrument (Applied Biosystems). Standard curves were plotted using serial dilutions of cDNA prepared from cells grown in iodate. The slopes of standard curves were used to calculate PCR efficiency according to the following equation:


$$E\;\left(\%\right)=\left[10^{\left(-1/\mathrm{slope}\right)}–1\right]\times 100$$

*E* values for all genes and primer pairs ranged from 95 to 105%, with *R*^2^ values >0.99.

### 2.6 Analytical technique

Iodate and iodide concentrations were determined using the ion chromatograph IC-2010 (Tosoh, Tokyo, Japan), as previously described (Yamazaki et al., 2020). The detection limits of iodate and iodide were 3 and 5 μM, respectively.

## 3 Results

### 3.1 Iodate reduction by marine aerobic bacterial cultures

**Figure 1:**
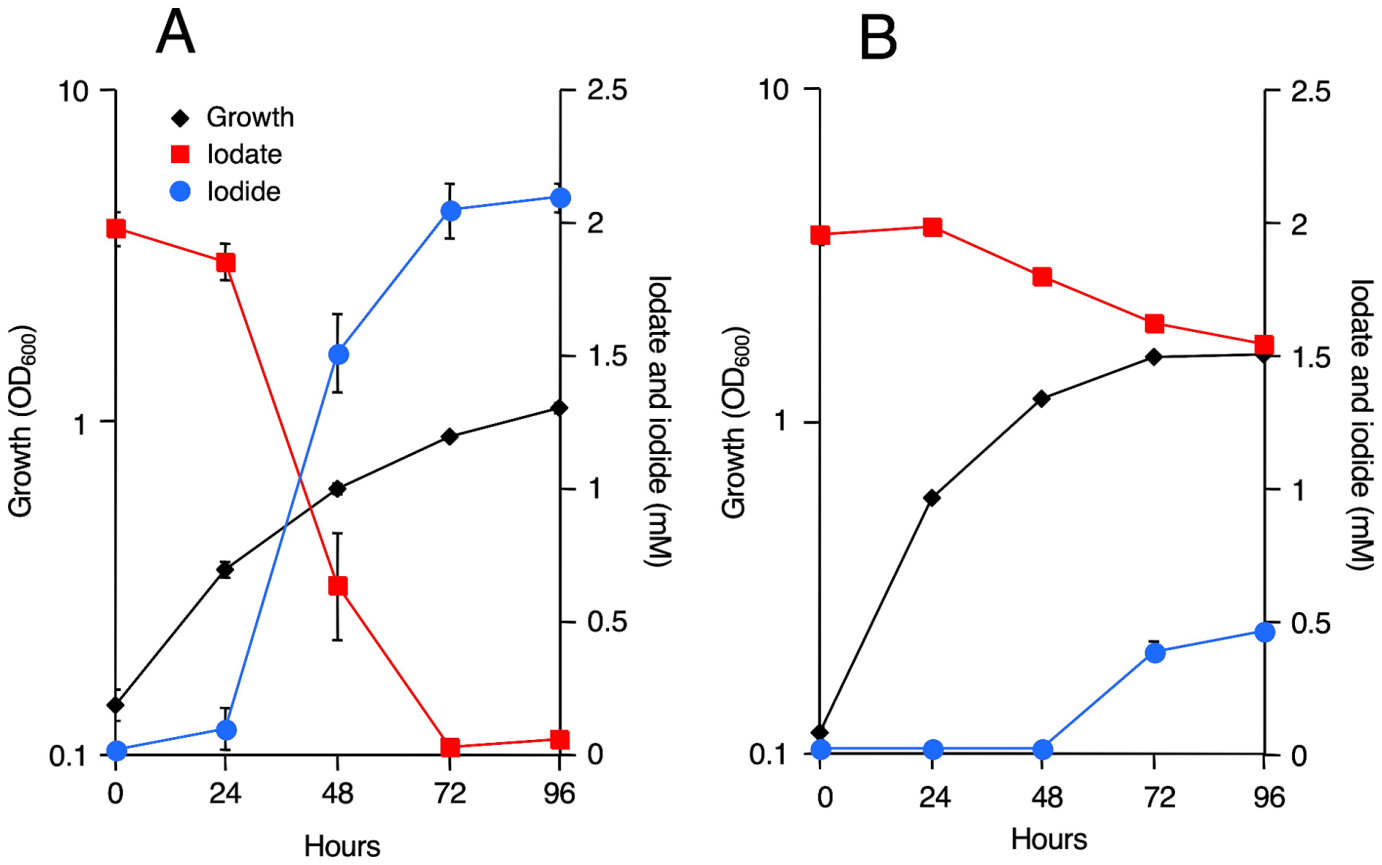
Iodate reduction by *Roseovarius azorensis* in marine broth 2216 under static **(A)** and shaking **(B)** conditions. All experiments were performed in triplicate, and error bars represent the standard deviations. Absence of bars indicates that the error is too small to be denoted by symbols.

**Figure 2:**
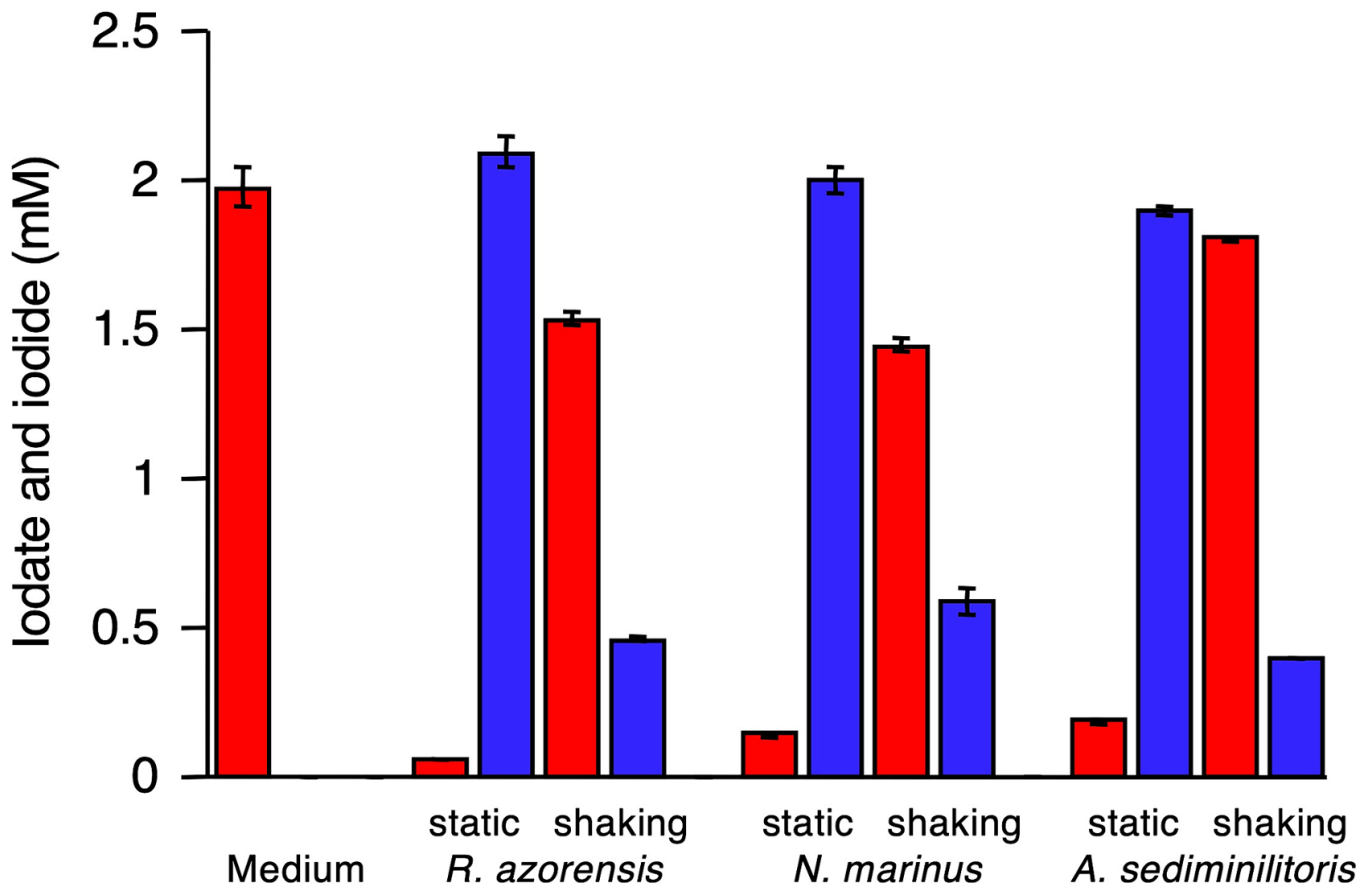
Iodate reduction by *R. azorensis*, *Notoacmeibacter marinus*, and *Aliiroseovarius sediminilitoris* in marine broth 2216 under static and shaking conditions. Iodate (red) and iodide (blue) concentrations after cultivation for 96 h are shown. Data from an uninoculated medium used as a control are also shown. All experiments were performed in triplicate, and error bars represent the standard deviations.

*R. azorensis*, *N. marinus*, and *A. sediminilitoris* were grown in the presence of 2 mM iodate under static and shaking conditions. Under static conditions, all bacteria reduced 2 mM iodate to iodide almost completely within 3 days (Figures 1, 2; Supplementary Figures S2, S3). However, the bacteria only reduced 0.1–0.5 mM iodate under shaking conditions despite much faster growth than that under static conditions. We attempted to grow these bacteria under anaerobic conditions in serum bottles filled with N_2_ gas. However, they could not grow or reduce iodate under these conditions (data not shown).

### 3.2 Iodate reduction by the washed cell suspension of *Roseovarius azorensis*

**Figure 3:**
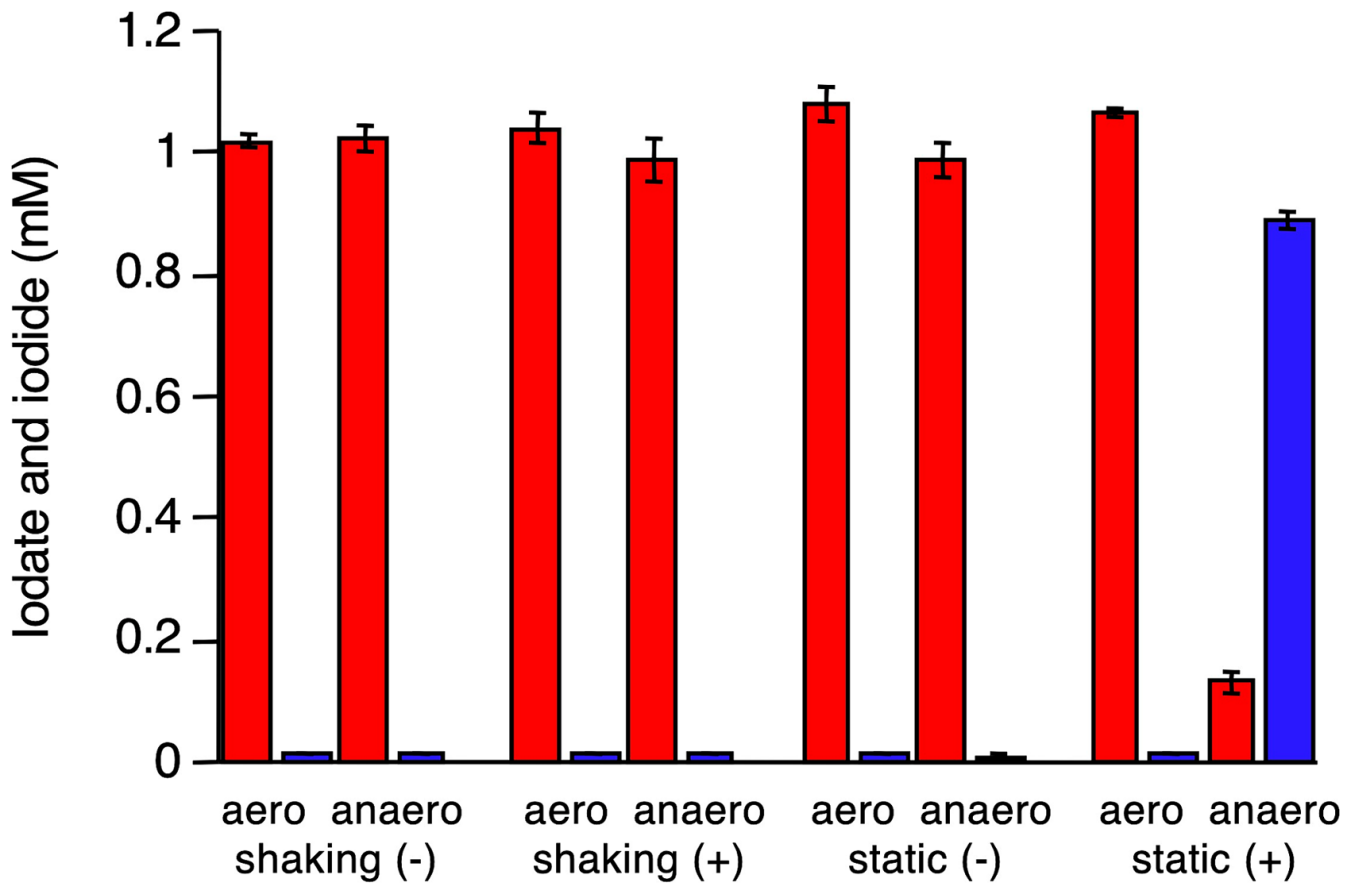
Iodate reduction by the washed cell suspension of *R. azorensis* under various conditions. Cells were pre-grown under shaking and static conditions with (+) or without (−) iodate. Then, the washed cells were incubated aerobically (aero) or anaerobically (anaero). Iodate (red) and iodide (blue) concentrations in washed cells after incubation for 8 h are shown. All experiments were performed in triplicate, and error bars represent the standard deviations.

*R. azorensis* was selected as the representative bacterium and grown under static and shaking conditions in the presence or absence of iodate. Washed cell suspensions were prepared from four types of cultures and incubated with iodate aerobically (in Erlenmeyer flasks with shaking) or anaerobically (in serum bottles filled with N_2_ gas). As shown in Figure 3, cells pre-grown under shaking conditions with or without iodate did not reduce iodate, regardless of the incubation conditions (aerobic or anaerobic). Similarly, cells pre-grown under static conditions without iodate did not reduce iodate, regardless of the incubation conditions. Notably, cells pre-grown under static conditions with iodate rapidly reduced iodate and produced iodide under anaerobic conditions (Figure 3). However, the same cells did not reduce iodate under aerobic conditions. These results suggest that the iodate-reducing capacity is induced only when *R. azorensis* is grown statically in the presence of iodate and that the induced cells can reduce iodate only under anaerobic conditions, but not under aerobic conditions.

### 3.3 Activity and identification of Idr

**Figure 4:**
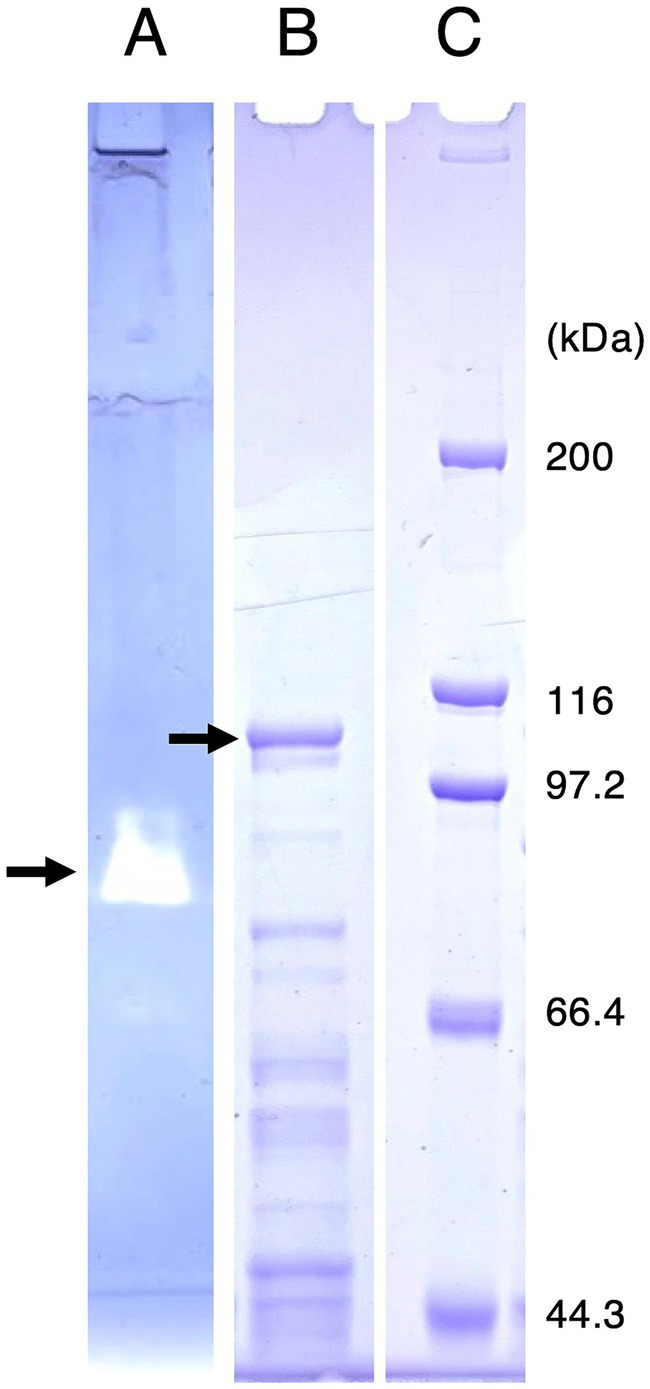
**(A)** Activity staining of iodate reductase (Idr) protein. *Roseovarius azorensis* crude extract was subjected to sodium dodecyl sulfate (SDS)-polyacrylamide gel electrophoresis (PAGE) at 4°C under non-denaturing conditions (without SDS and reducing agents). After electrophoresis, the gel was stained anaerobically with methyl viologen and iodate as the electron donor and acceptor, respectively. A clear band (arrow) represents the active band. **(B)** Separation of the excised active band under completely denaturing conditions (100°C, 5 min with SDS and 2-mercaptoethanol). The gel was stained with Coomassie brilliant blue R-250. A band with a molecular weight of approximately 100 kDa (arrow) was excised, trypsin-digested, and subjected to liquid chromatography-tandem mass spectrometry (LC-MS/MS) analysis. **(C)** Standard marker proteins. Note that **(B,C)** show images of the same SDS-PAGE gel.

*R. azorensis* was grown under static conditions in the presence of iodate, and Idr activity in the crude extract was determined using reduced MV as an electron donor. However, significant Idr activity of more than 0.1 U mg^−1^ was not detected in this study (data not shown). To visualize the Idr activity directly on a polyacrylamide gel, the crude extract was subjected to SDS-PAGE under non-denaturing conditions. The gel was stained for Idr activity under anaerobic conditions using reduced MV as an electron donor. A clear band was observed in the gel (Figure 4A), excised, and subjected to SDS-PAGE under denaturing conditions. As shown in Figure 4B, multiple bands with apparent molecular weights of 44–100 kDa were observed after Coomassie brilliant blue staining. A band with a molecular weight of approximately 100 kDa was excised, trypsin-digested, and subjected to liquid chromatography–tandem mass spectrometry (LC-MS/MS) analysis. From this band, 24 proteins with apparent molecular weights of 9–101 kDa were identified (Supplementary Table S1). One protein with accession number WP_093034476.1 showed the highest sequence coverage of 78% and highest number of unique protein peptides (Table 1). BLASTP analysis revealed that this protein was a homolog of IdrA in *Pseudomonas* sp. SCT, suggesting its involvement in iodate reduction in *R. azorensis*.

**Table 1:** The top 5 proteins identified from band in LC-MS/MS analysis.^a^

Protein name (gene name)	Accession number	Predicted molecular weight (kDa)	Number of protein unique peptides	Sequence coverage (%)
Iodate reductase large subunit (*idrA*)	WP_093034476.1	99.4	57	78
Formate dehydrogenase subunit alpha (*fdhF*)	WP_093032738.1	101.3	47	58
Aconitate hydratase (*acnA*)	WP_093034129.1	98.9	36	58
Protein translocase subunit SecA (*secA*)	WP_093035233.1	99.9	13	19
Translation initiation factor IF-2 (*infB*)	WP_093035251.1	89.1	3	7

a All proteins are listed in descending order of the number of protein unique peptides.

### 3.4 Transcriptional analysis of *idr* gene cluster

**Figure 5:**
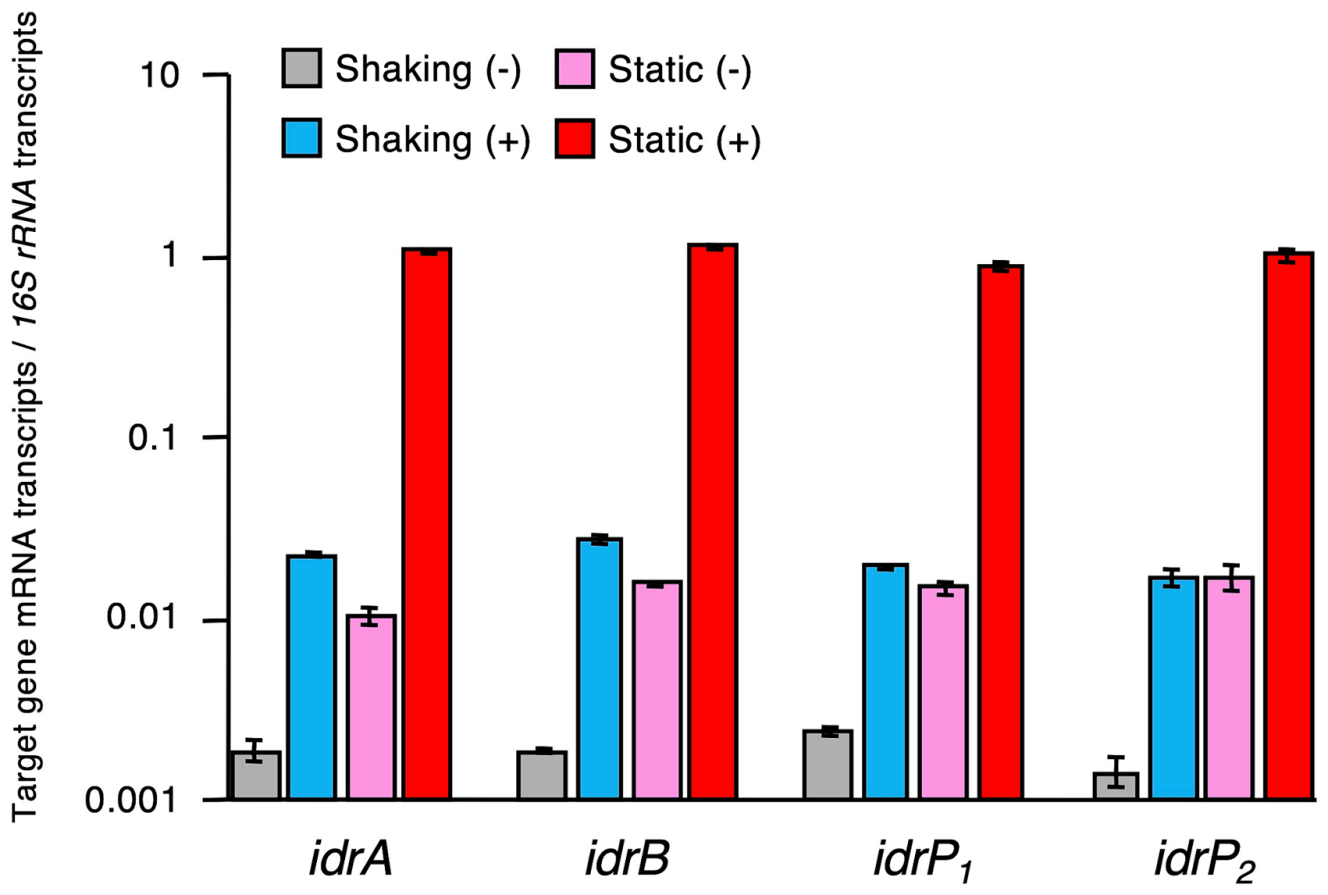
Expression levels of iodate reductase (*idr*) genes relative to those of *16S rRNA* gene in *R. azorensis* cells grown under various conditions. *R. azorensis* cells were grown under shaking conditions without (gray) or with (cyan) iodate and under static conditions without (pink) or with (red) iodate. Values represent the ratio of the relative quantity of *idrABP_1_P_2_* transcripts to that of *16S rRNA* gene transcripts. All values represent the mean values obtained from triplicate experiments, and bars represent the standard deviations.

Expression levels of *idrA*, *idrB*, *idrP_1_*, and *idrP_2_* relative to those of *16S rRNA* gene were quantified and compared using RNA extracted from cells grown under static and shaking conditions in the presence or absence of iodate. As shown in Figure 5 and Supplementary Table S2, expression patterns of these four genes were very similar. Highest expression was observed in the cells grown statically with iodate and lowest expression was observed in the cells grown under shaking conditions without iodate. The presence of iodate induced *idr* gene expression by 8.0–14-fold under shaking growth conditions and by 59–102-fold under static growth conditions (Supplementary Table S2). Static growth conditions induced *idr* gene expression by 5.7–12-fold in the absence of iodate and by 43–61-fold in the presence of iodate. Therefore, transcription of the *idrABP_1_P_2_* cluster is upregulated when *R. azorensis* is grown statically in the presence of iodate.

**Figure 6:**
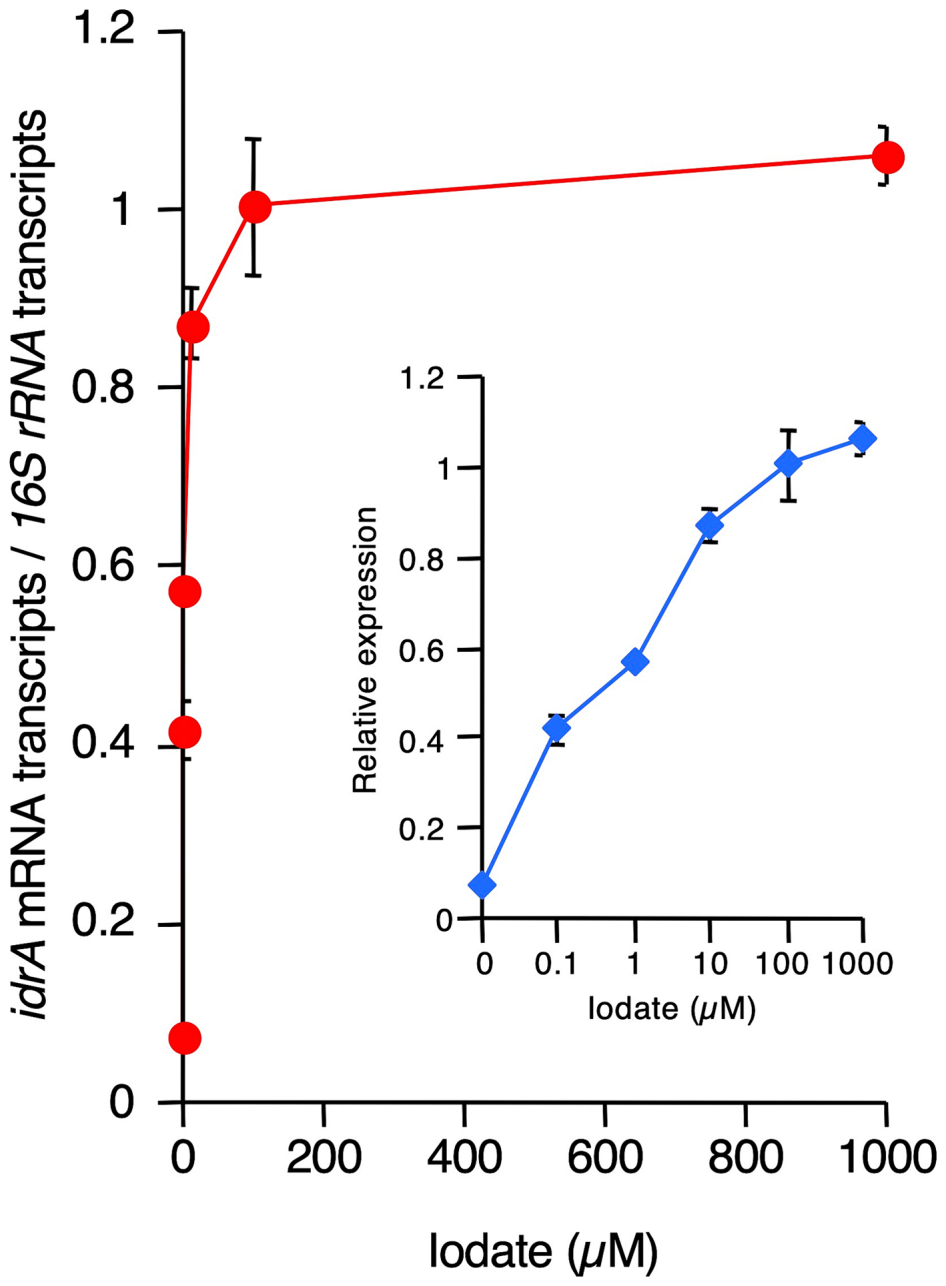
Effects of increasing concentrations of iodate on *idrA* expression levels in *R. azorensis*. Expression of *idrA* gene relative to that of *16S rRNA* gene was determined as a function of increasing iodate concentrations in *R. azorensis* cultures. The bacterium was grown statically in the absence (0 μM) or presence of the indicated concentrations of iodate. All values represent the mean values obtained from triplicate experiments, and bars indicate the standard deviations. Absence of bars indicates that the error is too small to be denoted by symbols. Inlet shows the same graph, but with the *X*-axis displayed in the logarithmic scale.

Next, we determined the effect of iodate on *idrA* expression. *R. azorensis* was grown under static conditions in the absence or presence of 0.1–1,000 μM iodate. As shown in Figure 6, *idrA* expression was detected at 0.1 μM iodate and was up to 14-fold higher than that in the non-iodate control.

## 4 Discussion

All three strains used in this study reduced 2 mM iodate almost completely under static conditions, but not under shaking conditions (Figures 1, 2; Supplementary Figures S2, S3). This was expected as the proteins encoded by *idrA* and *idrB* genes contain [3Fe–4S] and [2Fe–2S] clusters, respectively (Yamazaki et al., 2020). Fe–S clusters in metalloproteins are highly sensitive to molecular oxygen due to the decreased bioavailability of ferric irons and direct oxidation of these clusters by reactive oxygen species (Boyd et al., 2014). Experiments with washed cell suspension clearly showed that static growth conditions were necessary to confer iodate-reducing capacity to *R. azorensis* cells (Figure 3). Even though the cells exhibited iodate-reducing capacity, they did not reduce iodate under aerobic conditions, suggesting that enzyme Idr is oxygen-sensitive. Furthermore, transcriptomic analysis revealed that the *idrABP_1_P_2_* cluster was highly upregulated under static conditions (Figure 5). These results suggest that oxygen-limited conditions are essential for both iodate reduction by Idr and biosynthesis of Idr.

In addition to oxygen, iodate is another environmental factor significantly affecting iodate reduction by marine aerobic bacteria (Figures 3, 5). Induction of Idr and upregulation of *idrABP_1_P_2_* cluster expression by iodate have been observed in various iodate-reducing bacteria, such as *Pseudomonas* sp. SCT and *Azoarcus* sp. DN11 (Yamazaki et al., 2020; Sasamura et al., 2023). Therefore, iodate may be a universal inducer of the *idr* gene cluster in bacteria. Here, transcription of *idrA* was stimulated at iodate concentrations >0.1 μM (Figure 6). As the average concentration of total dissolved iodine in seawater is 0.45 μM (Wong, 1991) and iodate is the more stable form of iodine in seawater, transcription of *idr* gene cluster and biosynthesis of Idr proteins may be induced by environmental levels of iodate in seawater. Thus, aerobic bacteria may actually reduce iodate in marine surface waters at relatively low levels of oxygen.

Where are such conditions present in the well-oxygenated surface seawater? Although we did not use any devises for measurement of dissolved oxygen concentration in our culture medium, a recent paper by Huang et al. (2021) reported that dissolved oxygen concentrations in bacterial shaking and static cultures are approximately 8 mg L^−1^ and 1 mg L^−1^, respectively. Considering that typical dissolved oxygen concentrations in surface seawater are 5 to 8 mg L^−1^ (Wright et al., 2012), our shaking cultures successfully mimicked surface water environment. However, dissolved oxygen concentrations in surface seawater are sometimes very low, for example 1 to 3 mg L^−1^ in the eastern tropical North Pacific (Hoogakker et al., 2018). In addition, the interior of organic-rich aggregates or detritus could potentially be suitable micro-habitats for aerobic iodate-reducing bacteria. Heterotrophic aerobic bacteria associated with particulate organic matter may reduce iodate after active degradation and assimilation of organic compounds, which is combined with the rapid consumption of dissolved oxygen in the micro-habitats. Such local and transient hypoxic environments may be hot spots of iodate reduction in marine surface waters. If the associated particulate organic matter is microalgal remains, the iodate reduction observed during algal senescence may partly be due to heterotrophic aerobic bacteria (Bluhm et al., 2010; Hepach et al., 2020).

Putative dissolved oxygen concentration in our static cultures (1 mg L^−1^) was similar to those observed in OMZs, which occur in the Pacific Ocean, the Atlantic Ocean, and the Arabian Sea (Wright et al., 2012). Thus, it may be possible that aerobic bacteria also contribute to iodide production in these oxygen-depleted marine waters. It was reported that Alphaproteobacteria are predominant together with Gammaproteobacteria in OMZs of the eastern tropical South Pacific (Stevens and Ulloa, 2008) and the Arabian Sea (Bandekar et al., 2018). In addition, Kalvelage et al. (2015) found that Alphaproteobacterial terminal respiratory oxidase genes and transcripts are abundant in the metagenomes and metatranscriptomes of OMZs off Peru and Chile, suggesting that microaerobic respiration is a major mode of organic matter degradation in these OMZs. Furthermore, metagenomic analysis of the eastern tropical Pacific and the Arabian Sea OMZs actually identified *idrA* genes (Reyes-Umana et al., 2022a). These results suggest the possibility that aerobic iodate-reducing bacteria are producing iodide from iodate in various OMZs together with anaerobic iodate-respiring bacteria. Considering that total iodine concentrations in OMZs are sometimes significantly higher than those in surface seawater (Chapman, 1983), and that iodide is predominantly observed over iodate in these oxygen-depleted waters (Farrenkopf et al., 1997; Farrenkopf and Luther, 2002), aerobic bacteria might play a significant role in iodine cycling in deep waters.

Physiological mechanisms by which marine aerobic bacteria reduce iodate remain unclear. As these bacteria were unable to grow anaerobically with iodate as the terminal electron acceptor (data not shown), it may not be an anaerobic respiratory process. As iodate reduction only occurs under oxygen-limited conditions, iodate may be used as an electron sink to dispose the excess reducing power instead of oxygen. This iodate-reducing capacity may aid aerobic bacteria in maintaining their metabolism in iodate-rich seawater, even under oxygen-limited conditions, and might prevent them from losing viability. Considering that the *idrABP_1_P_2_* cluster is acquired by horizontal gene transfer (Reyes-Umana et al., 2022a, 2022b), iodate reduction should have an advantage for aerobic bacteria.

## Data Availability

The datasets presented in this study can be found in online repositories. The names of the repository/repositories and accession number(s) can be found in the article/Supplementary material.
