# Supplementary material for: Iodate reduction by marine aerobic bacteria
Source: Front Microbiol. 2024 Sep 18;15:1446596. doi: 10.3389/fmicb.2024.1446596 (PMC11445184; doi:10.3389/fmicb.2024.1446596)
Supplement: Supplementary file 2 [file Table_2.docx]

**Table S2.** Relative expression of the *idr* cluster genes in *Roseovarius azorensis* grown under various conditions

|  | Shaking (-)^a^ | Shaking (+)^b^ | Static (-)^c^ | Static (+)^d^ |
| --- | --- | --- | --- | --- |
| *idrA* | 1.00^e^ | 12.1 | 5.67 | 579 |
| *idrB* | 1.00^e^ | 14.4 | 8.42 | 617 |
| *idrP_1_* | 1.00^e^ | 8.03 | 6.15 | 361 |
| *idrP_2_* | 1.00^e^ | 11.7 | 12.0 | 717 |

^a^ *R. azorensis* was grown under shaking conditions without iodate.

^b^ *R. azorensis* was grown under shaking conditions with iodate.

^c^ *R. azorensis* was grown under static conditions without iodate.

^d^ *R. azorensis* was grown under static conditions with iodate.

^e^ Relative expression under shaking conditions without iodate was regarded as equivalent to 1.00.
