# Supplementary material for: Iodate reduction by marine aerobic bacteria
Source: Front Microbiol. 2024 Sep 18;15:1446596. doi: 10.3389/fmicb.2024.1446596 (PMC11445184; doi:10.3389/fmicb.2024.1446596)
Supplement: Supplementary file 3 [file Table_3.docx]

**Table S3.** Primers used for qRT-PCR assays

| Target gene |  | Sequence (5’ to 3’) | Amplicon size (bp) | *E* (%) | *R*^2^ |
| --- | --- | --- | --- | --- | --- |
| *16S rRNA* | Forward | AACGAGCGCAACCCACA | 85 | 98 - 104 | 0.997 – 0.999 |
|  | Reverse | CACCTTCCTCCCGCTTATCAC |  |  |  |
| *idrA* | Forward | GGGAAATCGAGAGCGGCTA | 112 | 101 | 0.998 |
|  | Reverse | TTGGCGATGAATGTCTGGAA |  |  |  |
| *idrB* | Forward | CGCAGGAACTGGTTTCGTC | 101 | 102 | 0.999 |
|  | Reverse | GTCGTCATTGGGATAGGTAAAGGT |  |  |  |
| *idrP_1_* | Forward | GCATGGATGAGGCGACCTA | 87 | 101 | 0.998 |
|  | Reverse | TGCGGAACTTGCCCTTG |  |  |  |
| *idrP_2_* | Forward | TTATGTCGTGACCAAGGACGAG | 102 | 105 | 0.997 |
|  | Reverse | CCAAACACGCCCGAATG |  |  |  |
