# Supplementary material for: Iodate reduction by marine aerobic bacteria
Source: Front Microbiol. 2024 Sep 18;15:1446596. doi: 10.3389/fmicb.2024.1446596 (PMC11445184; doi:10.3389/fmicb.2024.1446596)
Supplement: Supplementary file 4 [file Presentation_1.pdf]

Supplementary Material

1 Supplementary Figures

Supplementary Figure 1. Distribution of *idrA* gene among marine aerobic bacteria. Neighbor-joining phylogenetic analysis was conducted using MEGA11. Blue and green letters indicate *idrA* from Alpha- and Gammaproteobacteria, respectively. Bacterial strains used in this study are represented as blue bold letters. The *idrA* genes of iodate-respiring bacteria isolated to date are also indicated in red letters. Note that *idrA* genes from facultative anaerobic bacteria such as *Vibrio* spp. and *Pseudovibrio* spp. are eliminated from this figure.

Supplementary Figure 2. Iodate reduction by *Notoacmeibacter marinus* in marine broth 2216 under static (**A**) and shaking (**B**) conditions. All experiments were performed in triplicate, and error bars represent the standard deviations. Absence of bars indicates that the error is too small to be denoted by symbols.

Supplementary Figure 3. Iodate reduction by *Aliiroseovarius sediminilitoris* in marine broth 2216 under static (**A**) and shaking (**B**) conditions. All experiments were performed in triplicate, and error bars represent the standard deviations. Absence of bars indicates that the error is too small to be denoted by symbols.

2 Supplementary Tables
