# Supplementary figures and images for: Iodate reduction by marine aerobic bacteria
Source: Front Microbiol. 2024 Sep 18;15:1446596. doi: 10.3389/fmicb.2024.1446596 (PMC11445184; doi:10.3389/fmicb.2024.1446596)

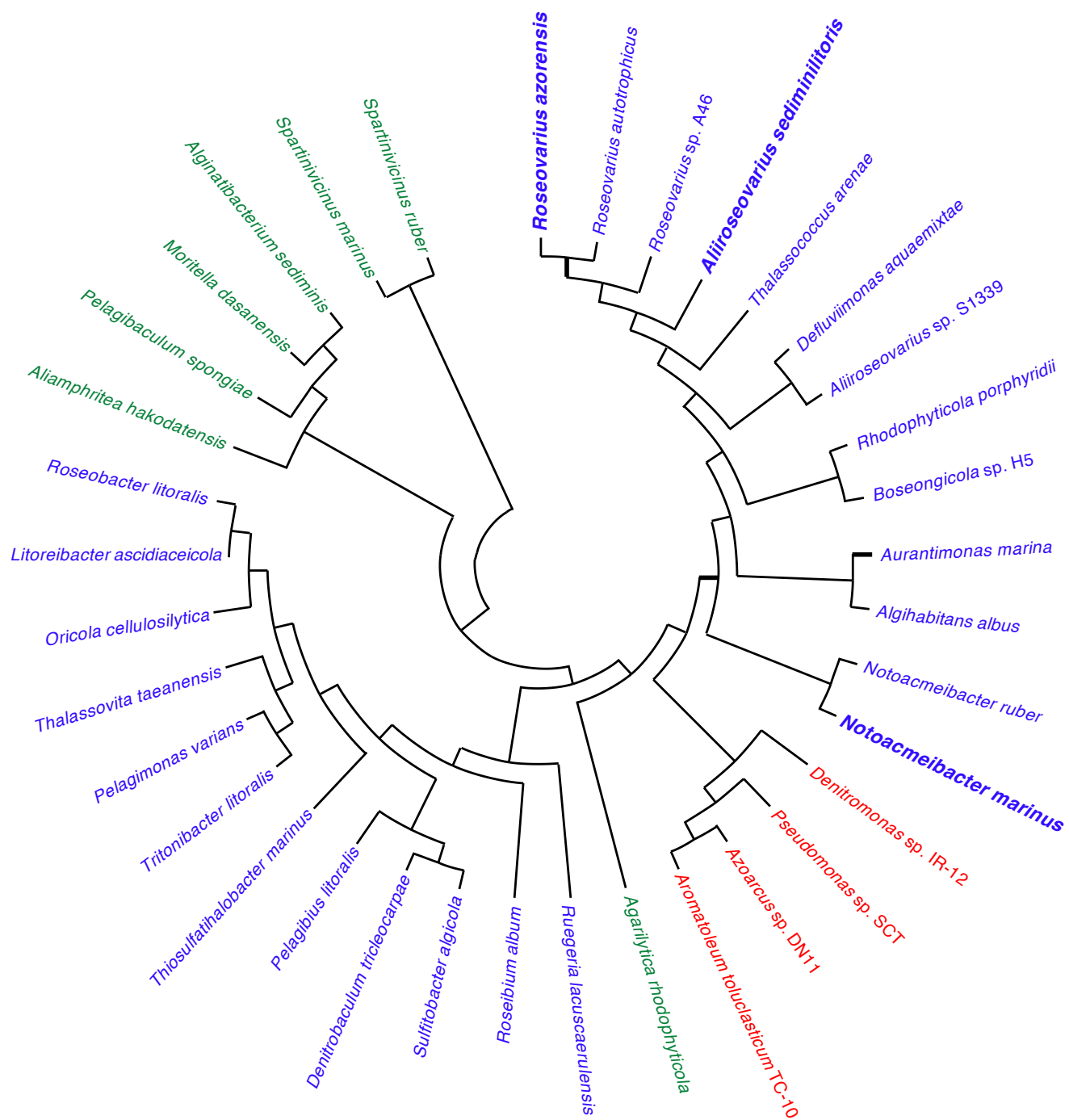

Fig. S1. Kine et al.

Supplement: Supplementary file 5 [file Data_Sheet_1.PDF]

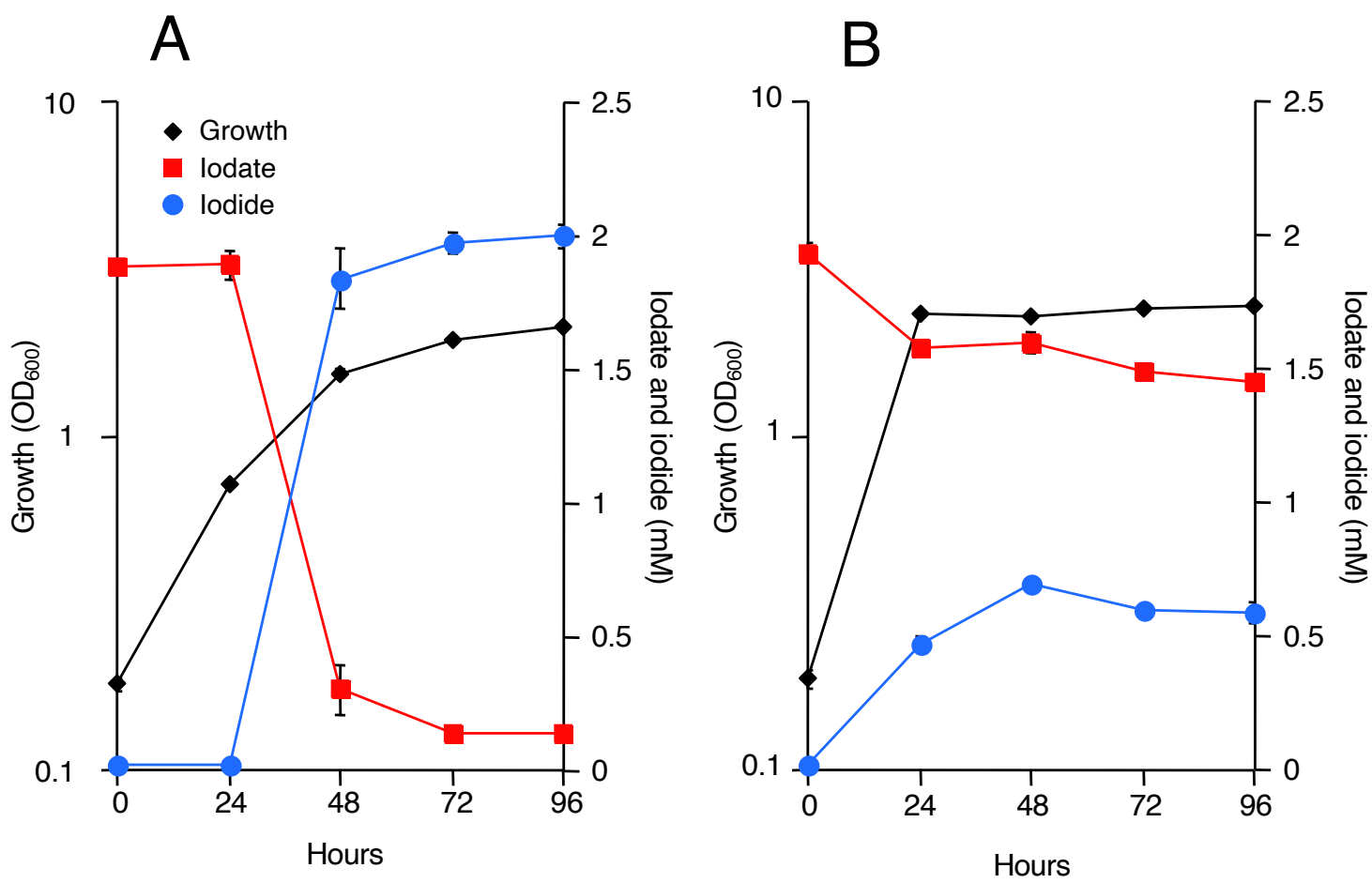

Fig. S2. Kine et al.

Supplement: Supplementary file 6 [file Data_Sheet_2.PDF]

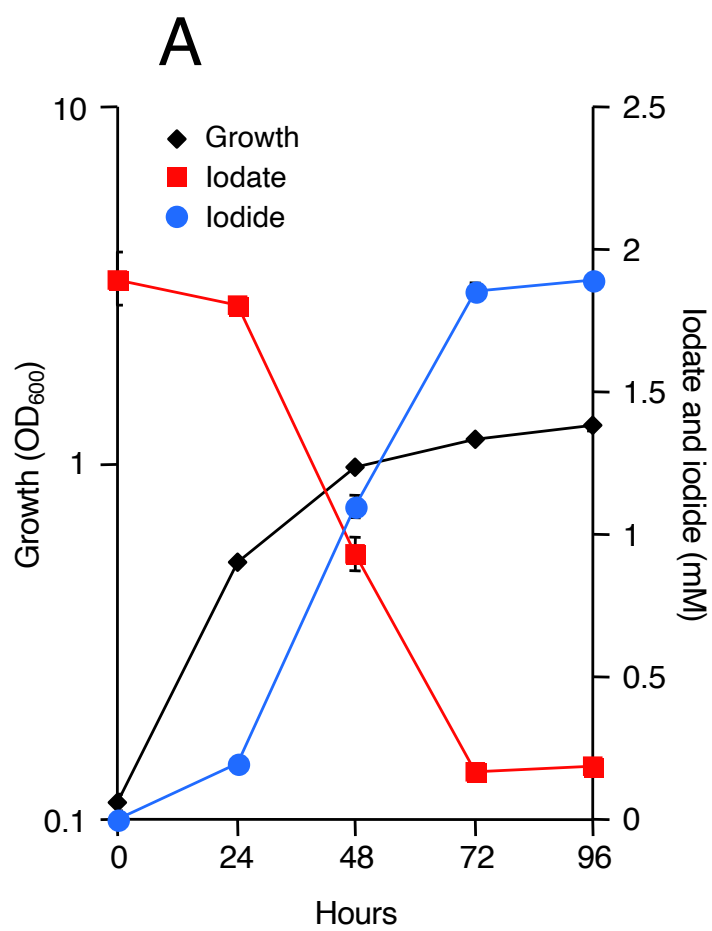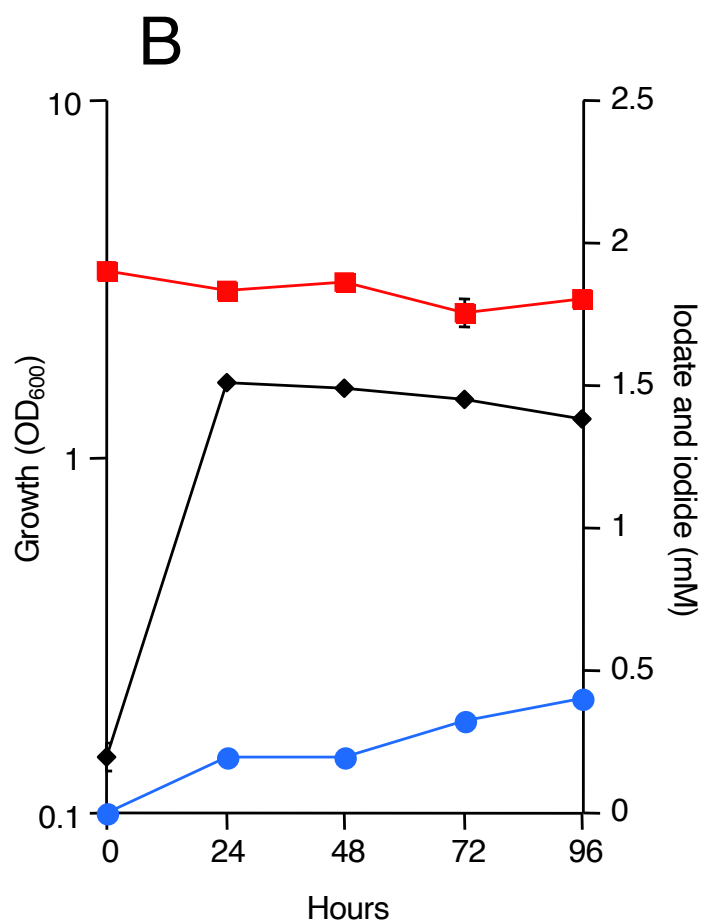

Fig. S3. Kine et al.

Supplement: Supplementary file 7 [file Data_Sheet_3.PDF]
